# Energy-Saving Analysis of Hydraulic Hybrid Excavator Based on Common Pressure Rail

**DOI:** 10.1155/2013/560694

**Published:** 2013-09-30

**Authors:** Wei Shen, Jihai Jiang, Xiaoyu Su, Hamid Reza Karimi

**Affiliations:** ^1^School of Mechatronics Engineering, Harbin Institute of Technology, Harbin 150080, China; ^2^College of Automation, Harbin Engineering University, Harbin 150001, China; ^3^Faculty of Technology and Science, University of Agder, 4898 Grimstad, Norway

## Abstract

Energy-saving research of excavators is becoming one hot topic due to the increasing energy crisis and environmental deterioration recently. Hydraulic hybrid excavator based on common pressure rail (HHEC) provides an alternative with electric hybrid excavator because it has high power density and environment friendly and easy to modify based on the existing manufacture process. This paper is focused on the fuel consumption of HHEC and the actuator dynamic response to assure that the new system can save energy without sacrificing performance. Firstly, we introduce the basic principle of HHEC; then, the sizing process is presented; furthermore, the modeling period which combined mathematical analysis and experiment identification is listed. Finally, simulation results show that HHEC has a fast dynamic response which can be accepted in engineering and the fuel consumption can be reduced 21% to compare the original LS excavator and even 32% after adopting another smaller engine.

## 1. Introduction

The demand for fuel efficient and low-emission hydraulic excavators has been improved due to the increasing energy crisis and environmental deterioration recently [1, 2]. Hence, reducing the fuel consumption of excavators has become a hot topic for the manufacturers and researchers, and some useful conclusions are made until now [3–6]. The main methods include positive flow rate control [7], negative flow rate control and load sensing control systems [8–10], and so forth. Each system has its own characteristics; however, their basic theory is similar. Most of them adopt the use of multivalves to control the speed of actuators and by sensing the pilot system pressure to control the displacement of the main pump so as to make the output power of engine match the load power. So, these systems can reduce overflow loss. They are always combined with constant power control for the engine in order to avoid the engine shutdown; for example, the pump should reduce the displacement when the output power exceeds the setting power. But the common disadvantages of these systems are huge metering losses and an inability to recover energy. The first is because multi-valves are using throttling to control flow rate. The other one is due to the aiding energy being dissipated in heat during excavator working cycle. For example when the swing is braking or the boom is lowering during every cycle, the braking energy and the gravitational energy are wasted by converting into heat. One more drawback is that it needs a large cooling system to decrease system temperature, but it deteriorates the energy consumption more. Since there is the energy recuperation potential, especially hybrid technology which is already used widely in the hybrid vehicle [11–13], hybrid excavators that make use of batteries or capacitors may be used [14–17]. For example the parallel architecture from Komatsu Company [18, 19], output power from engine is balanced by using the electric motor/generator. Furthermore, the swing hydraulic motor is replaced by an electric motor which can eliminate the metering loss for the swing system. Braking and gravitational energy can be converted to electrical energy and stored in a battery, and the energy can be used in the next cycle by converting to hydraulic energy again. Later, some famous excavator manufacturers such as Kobelco [20], Hitachi, and Sony [21] introduced their own electric hybrid prototype, and in these settings, series and even series-parallel configuration are used. An electric hybrid excavator is a good taste for reducing the fuel consumption because it can eliminate throttling loss in some range and make energy recuperation possible. But it needs to be noticed that an electric hybrid excavator consists of hydraulic and electric actuators, and the energy needs to be converted among mechanical, hydraulic, and electric energy which will result in low efficiency; besides, adding electric actuators is difficult for modifying based on the existing manufacturing process. Moreover, it does not cancel the multi-valves as flow control component for the cylinder, so the metering loss remains. One alternative for hybrid excavators is hydraulic hybrid excavator based on common pressure rail (HHEC) adopting hydraulic accumulators as the storing component [22–24]. It not only eliminates the theoretical metering loss but can also recover energy. In particular, the most significant advantage of using hydraulic accumulators is the seamless interface (hydraulic energy) during recovering and reusing, so the efficiency of the circuit is high.

## 2. Machine Description

**Figure 1:**
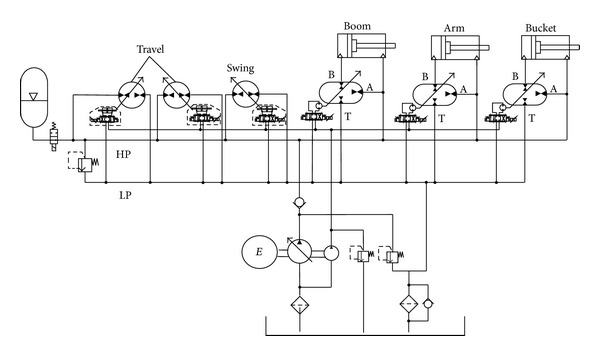
Simplified schematic of HHEC.

**Figure 2:**
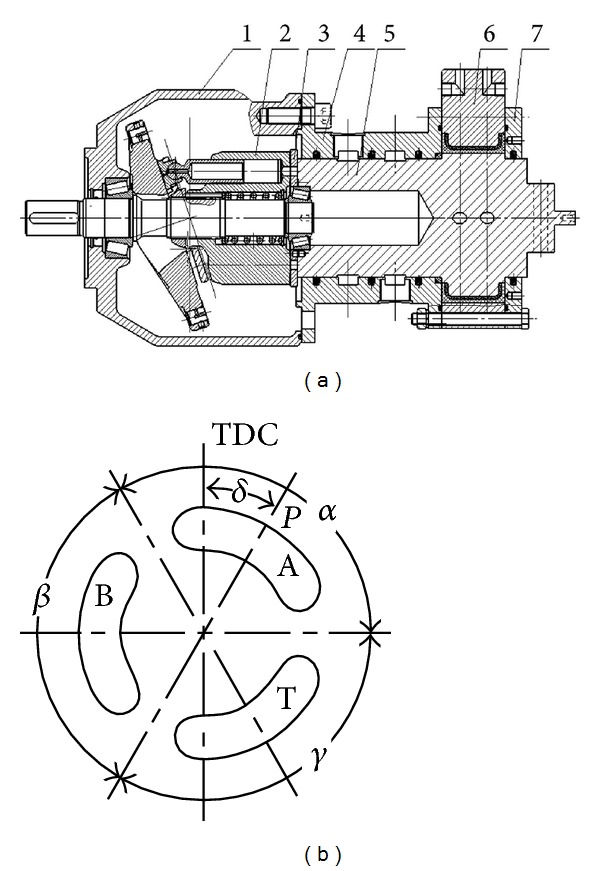
Structure of SHT. (1) Case of original pump. (2) Block of original pump. (3) New port plate. (4) Case of swing motor. (5) Rotor of swing motor. (6) Stator of swing motor. (7) Rear cover of swing motor.

The simplified schematic of HHEC is shown in Figure 1 [25, 26]. Common pressure rail (CPR) is composed of high pressure pipeline and low pressure pipeline, which is similar to the grid system that is composed of high-voltage line and low-voltage line [27]. Electric equipment is connected to the grid in parallel. In CPR, the constant pressure variable pump and hydraulic accumulator constitute the high pressure oil sources, and multiple different loads connect in parallel between the high pressure and the low pressure pipeline, in which rotational load and linear load are driven by a motor/pump and a cylinder, respectively. When the load changes, the pressure of system has a small fluctuation while the flow varies with the load, and the load can be adapted by regulating the displacement of the hydraulic pump/motor. The 4-quarter working principle of motor/pump can make energy recovery possible. For the cylinder, that is hard to change displacement normally, the hydraulic transformer (HT) is used to control the cylinder. Traditional hydraulic transformers can accomplish the aim of transforming from the hydraulic constant pressure end to the load end, but it is massive, expensive, and has low efficiency. The new HT merges two components connected with the shaft into one component. There are three ports in the port plate which is linked with the high pressure line, the load, and the tank, respectively. And the pressure is varied by regulating the angle of the port plate [28, 29].  Figure 2(a) shows the structure of the swash plate type hydraulic transformer (SHT) which is modified by axial piston pump, and it is the second HT at Harbin Institute of Technology [30, 31]. The whole HT can be divided into two parts which are axial piston pump and swing motor. The former port plate is changed, and Figure 2(b) shows the section of port plate [29], which is also rigidly fixed with the swing motor, and in this picture, **α**,  **β**  and  **γ**  are the length of the A, B, and T ports, respectively. So, we can control the motor rotation angle by turning the port plate angle. Both swash plate of motor/pump and the port plate of SHT are controlled by servo valves. Because the flow rate (10 L/min) is so little, the energy consumption can be accepted. 

## 3. Sizing of HHEC

It is noticed that this paper is to compare the fuel consumption between HHEC and the original systems during one digging cycle. The digging cycle includes an excavator digging a bucket of dirt, rotating and releasing the load onto a pile and then returning to its initial position. It can be found that the travel system is not involved in the cycle, so the sizing process and simulation will not include it. The design assumption is decided that the original hydraulic cylinders would maintain their original dimensions. This decision is useful for comparing the original system, and it will simplify the development of a prototype machine. In future work, the travel system can be integrated easily, so that we can use SHTs which are used for controlling the boom and stick to control travel motors because the travel system and other actuators are not usually used simultaneously [32]. 

### 3.1. Specifying the Accumulator

The maximum pressure of the hydraulic accumulator should be chosen lower than the maximum system pressure which also should be safe for all components in system. So we choose the maximum system pressure according to the original system,

(1) $$\begin{array}{c} p_{\text{acc},\max}\leq p_{\max}, \end{array}$$

where *p*_acc,max⁡_ is the maximum pressure of hydraulic accumulator; *p*_max⁡_ is the maximum system pressure.

Volume selection of the hydraulic accumulator should follow the rule that it is enough to absorb the braking and gravitational energy. The swing and boom are the two biggest potential; hence, the maximum recovery energy can be calculated by

(2) $$\begin{array}{c} E_{\max}=0.5\times \left(J_{\text{cabin}}\cdot \omega^{2}+m_{\text{eq}\_b}\cdot v^{2}\right), \end{array}$$

where *J*_cabin_ is the rotational inertia of cabin; *ω* is the average angular velocity of the swing mechanism; *m*_eq_*b*_ is the mass of boom; *v* is the boom speed. 

The volume of hydraulic accumulator is equal to

(3) $$\begin{array}{c} V_{a}={\left(\frac{p_{\text{pre}}}{p_{\min}}\right)}^{1/k}\cdot \frac{E_{\max}\cdot \left(k-1\right)}{p_{\min}\cdot {\left(p_{\min}/p_{\max}\right)}^{(1-k)/k}-p_{\min}}, \end{array}$$

where *p*_pre_ is the initial air pressure of the accumulator and *p*_min⁡_ is the minimum of system pressure; *k* is the gas changeful index.

### 3.2. Sizing the Volume of SHT

We define the relative volume which means that the three ports A, B, and T have their own volume with the rotation of the port plate. So the volumes are [29]

(4) $$\begin{array}{c} V_{\text{A}}=\frac{V_{\text{HT}}}{2\pi }\cdot \sin\frac{\alpha }{2}\cdot \sin\delta ,\\ V_{\text{B}}=\frac{V_{\text{HT}}}{2\pi }\cdot \sin\frac{\beta }{2}\cdot \sin\left(\delta -\frac{\alpha }{2}-\frac{\beta }{2}\right),\\ V_{\text{T}}=\frac{V_{\text{HT}}}{2\pi }\cdot \sin\frac{\gamma }{2}\cdot \sin\left(\delta +\frac{\alpha }{2}+\frac{\gamma }{2}\right), \end{array}$$

where *V*_HT_ is the total volume of SHT and *δ* is the rotation degree of port plate. 

**Figure 3:**
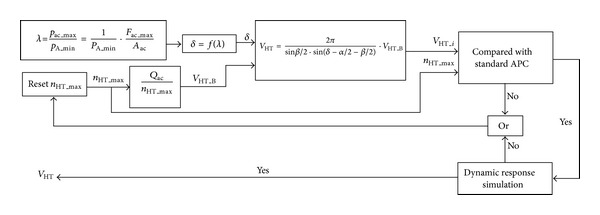
Flow chart of sizing SHT.

Equation (4) means that we can calculate the volume of the SHT if the *V*_B_ and *δ* are confirmed. We use iterative trial and error method to calculate the volume. The detailed flow chat is shown in Figure 3.

**Figure 4:**
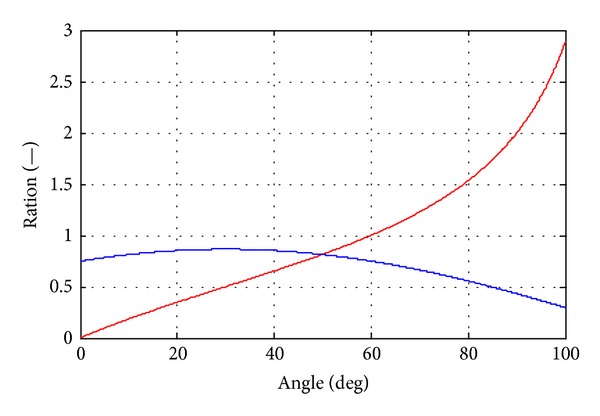
Curves of *V*_B_/*V*_HT_ and *λ* change corresponding to different values of *δ*.

Firstly, the transformer ratio *λ* can be determined through the maximum the force on cylinder and the minimum pressure of high pressure pipe line (PHP); then, *δ* can be obtained by (5) [29]. One constraint is needed, so that the reasonable range for *δ* is from 0 to 100 degrees [33]. Secondly, *V*_B_ can be obtained according to the cylinder speed; the maximum rotation speed should be assumed during this step. The final step is to check whether the numbers make sense. Because the SHT is modified from the swash plate axial piston pump, the standard document from manufacturers can be a criterion. If the numbers do not match the document, the sizing progress should go back to the setting of maximum rotation speed. Furthermore, since the sum torque for driving the block is generated from every port of the SHT, the rotation speed should change during the operation process. The dynamic response of rotation speed must be considered.  Figure 4 shows the relationship between *V*_B_/*V*_HT_ and *δ* with the blue curve, and the red curve represents the relation between *λ* and *δ* (the rotation speed is assumed constant). The results show that *V*_B_ reduces fast after *λ* is bigger than 1 and small values of *V*_B_ will result in slow response. Hence, after the volume and rotation speed are chosen, the dynamic response should also be obtained through simulation. The method can be found in [33]. And if the result does not meet the dynamic requirement, the sizing should also go back to the setting of maximum rotation speed until the parameters meet all demands, consider

(5) λ=pBpA=(−sin⁡(α2)·sinδ−(pTpA)·sin⁡(γ2) ·sin(δ+(α2)+(γ2)))×(sin⁡(β2)·sin(δ−(α2)−(β2)))−1.

### 3.3. Volume Sizing of the Main Pump

The main pump is one critical component for keeping the pressure of the high pressure line quasiconstant. So the main pump should supply enough flow for system. The critical parameter for main pump is volume, which should meet the two requirements in the aspects of flow rate and power:

(6) $$\begin{array}{c} V_{\text{main}}\geq \frac{\max\{Q_{\text{bo}}+Q_{\text{sw}}+Q_{\text{ar}}+Q_{\text{bu}},Q_{\mathrm{tr}}\}}{n_{E}}, \end{array}$$

where *Q*_bo_ is the maximum flow rate of boom, and *Q*_sw_, *Q*_ar_, *Q*_bu_, and *Q*_tr⁡_ represent the maximum flow rate of swing motor, arm cylinder, bucket cylinder, and travel motor, respectively,

(7) $$\begin{array}{c} V_{\text{main}}\leq \frac{P_{E}\cdot 2\pi }{n_{E}\cdot \left(p_{\text{acc},\max}-p_{\min}\right)}, \end{array}$$

where *P*_*E*_ is the engine power, *n*_*E*_ is engine rotation speed.

## 4. Modeling Process

A whole model which intergraded with mechanical, hydraulic, and control systems is needed to simulate the actuators dynamic response and fuel consumption. 

### 4.1. Mechanical Model of HEEC

**Figure 5:**
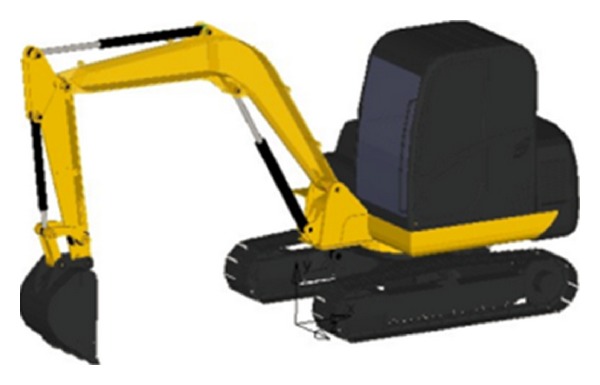
Mechanical model of HEEC.

The 3D solid mechanical model is constructed by Solidworks and input Simulation X; then, the material and density are also input.  Figure 5 shows the model.

### 4.2. Hydraulic Model of HEEC

#### 4.2.1. Engine Model

The dynamic response of engine is calculated by

(8) $$\begin{array}{c} n_{E}=\int \frac{1}{J_{E}}\left(u_{E}\cdot T_{E}-T_{f}-T_{L}\right)\text{d}t, \end{array}$$

where *u*_*E*_ is the throttle, *T*_*E*_ represents the maximum engine output torque, and *T*_*f*_ is the friction torque. The sum torque of load is presented by *T*_*L*_, and the initial of engine is *J*_*E*_.

*T*_*f*_ is estimated according to [34], and the equation is

(9) $$\begin{array}{c} T_{f}=\frac{V_{E}\times 10^{-3}}{12.5}\left(75000+\frac{48\times n_{E}}{1000}+0.4\times {\overset{-}{S}}_{p}^{2}\right), \end{array}$$

where ${\overset{-}{S}}_{p}$ is the piston speed.

The sum of torque load is equal to the sum of the main pump output torque and loss torque

(10) $$\begin{array}{c} T_{L}=\frac{p_{\max}\cdot V_{\text{main}}}{2\times \pi }+T_{\text{loss}}. \end{array}$$

#### 4.2.2. The Displacement Control System of Pump and SHT

**Figure 6:**
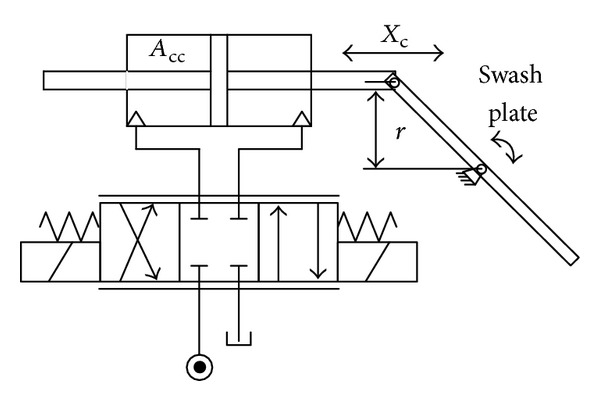
Displacement control mechanism of the main pump and motor/pump.

The common control architecture in HHEC is using servo valves to control the position of the cylinder or motor. For the main pump and motor/pump, Figure 6 shows the principle. For the SHT, the cylinder and swash plate are replaced by the motor and port plate, respectively. Hence, the model is similar. One assumption is also made that the pressure in the link tank is zero.

The dynamic characteristics of the servo valve are simplified as a 2nd-order transfer function, and the numbers are from catalog data. So the relation between output flow rate *Q*_sf_ of the servo valve and the input current *I* signal is

(11) $$\begin{array}{c} \frac{Q_{\text{sf}}\left(s\right)}{I\left(s\right)}=\frac{K_{v}}{\left(s^{2}/\omega_{\text{sf}}^{2}\right)+\left(2\xi_{\text{sf}}/\omega_{\text{sf}}\right)s+1}, \end{array}$$

where *K*_*v*_ is the flow gain of the servo valve; *ω*_sf_ and *ξ*_sf_ are natural frequency as well as damping ratio.

The pressure build-up in the high pressure chamber is calculated using the following equation:

(12) $$\begin{array}{c} {\dot{P}}_{k}=\frac{\beta_{e}}{V_{0\_c}+A_{\text{cc}}\cdot \left(x_{i}+x_{c}\right)}\left(q_{\text{sf}}-A_{\text{cc}}{\dot{x}}_{c}-c_{c\_i}\cdot P_{k}\right), \end{array}$$

where *β*_*e*_ represents bulk modulus of the fluid; *x*_*i*_ is the initial position of cylinder (in the middle); *V*_0_*c*_ is the hydraulic capacitance; *c*_*c*_*i*_ is the coefficient of internal leakage and the external leakage is ignored.

For the swing motor which drives the port plate, the pressure build-up equation is

(13) $$\begin{array}{c} {\dot{P}}_{k\_z}=\frac{\beta_{e}}{V_{0\_z}}\left(q_{\text{sf}}-D_{m}\cdot \dot{\delta }-\left(C_{z\_i}+C_{z\_e}\right)\cdot P_{k\_z}\right). \end{array}$$

In above equation, *D*_*m*_ is the swing motor volume, *C*_*z*_*i*_ and *C*_*z*_*e*_ are the internal and external leakage coefficients of the swing motor, respectively; *V*_0_*z*_ is also the hydraulic capacitance including the initial volume of motor and pipe.

A force balance is applied to the cylinder and results in the following:

(14) $$\begin{array}{c} A_{\text{cc}}p_{k}=m_{c}\frac{d^{2}x_{c}}{dt^{2}}+B_{c}\frac{dx_{c}}{dt}+k_{c}x_{c}+F_{\text{fc}}, \end{array}$$

where *m*_*c*_ represents the equivalent mass of cylinder module; *B*_*c*_ is the coefficient of viscous friction; *k*_*c*_ is the spring stiffness of central spring in the cylinder, and *F*_fc_ is the equivalent load which is applied to the cylinder.

The similar torque balance equation for the swing motor is calculated by

(15) $$\begin{array}{c} D_{m}\cdot P_{k\_z}=J_{m}\ddot{\delta }+B_{m}\cdot \dot{\delta }+K_{m}\cdot \delta +T_{t}, \end{array}$$

where *B*_*m*_ is the coefficient of viscous friction of swing motor; *δ* represents the swing angle of the swing motor (equal to angle of port plate of SHT); *K*_*m*_ is the coefficient of elasticity; *T*_*t*_ is the equivalent load torque; *J*_*m*_ is the equivalent moment of inertia of cylinder module. 

#### 4.2.3. The Efficiency Models of Main Pump and Motor/Pump

**Figure 7:**
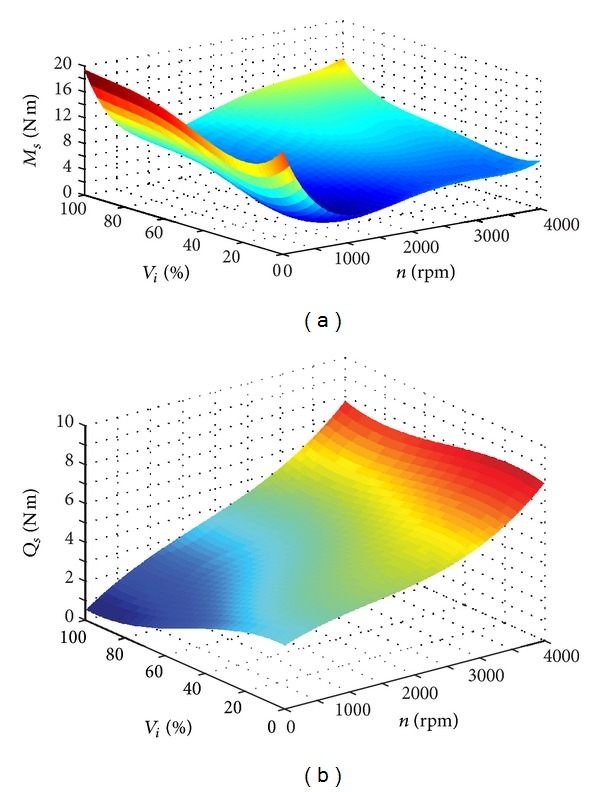
Torque loss and volumetric loss under different conditions. *Q*_*r*_ = *f*_*Q*_(*n*_*p*_, Δ*p*, *V*_*i*_), and *T*_*r*_ = *f*_T_(*n*_*p*_, Δ*p*, *V*_*i*_) when Δ*p* = 26 Mpa.

The main pump and motor/pump belong to the axial piston component and are very complicated to be described as mathematical equations. To make the model precise and easy to use, this paper adopts the method and date in [35]. An empirical model based on data from steady-state measurements is used to predict the complicated volumetric and torque loss characteristics, and the scale rule is also applied to simulate different sizes of axial piston components. A three-dimensional surface to volumetric or torque loss is created as a function of the pump pressure, rotation speed, and displacement. One condition is selected to show the losses in Figure 7. Consider

(16) f(nc,Δp,Vi)=a111·nc3+a112·nc2·Δp+a113·nc2·Vi+a11·nc2+a122·nc·Δp2+a123·nc·Δp·Vi+a12·nc·Δp+a133·nc·Vi2+a13·nc·Vi+a1·nc+a222·Δp3+a223·Δp2·Vi+a22·Δp2+a223·Δp·Vi2+a23·Δp·Vi+a2·Δp+a333·Vi3+a33·Vi2+a3·Vi+a0.

According to the above equation, we can calculate the real output flow and torque after inputing the three parameters: pump pressure, rotation speed, and displacement [35]. 

#### 4.2.4. The Subsystem of SHT Control Cylinder

The displacement control system is introduced in Section 4.2.2. The following part is focused on the SHT control cylinder and motor/pump control cabin. In the HHEC, the boom, arm, and bucket are all controlled by SHTs. They have the same modeling method. An arm subsystem is chosen for presenting the procedure because it behaves the most complicated working condition.

The block of the SHT is driven by the sum of toque which is generated by the three ports of the SHT. The sum of torque among three ports is as follows:

(17) $$\begin{array}{c} \Delta T=J_{\text{HT}}{\dot{\omega }}_{\text{HT}}=T_{\text{A}}+T_{\text{B}}+T_{\text{T}}, \end{array}$$

where *J*_HT_ is the moment of inertia of hydraulic transformer; *ω*_HT_ is angular speed of the block of SHT.

In the above equation, the torque of each port is followed:

(18) $$\begin{array}{c} T_{\text{A}}=\frac{p_{\text{A}}\cdot V_{\text{HT}}}{2\pi }\cdot \sin\frac{\alpha }{2}\cdot \sin\delta ,\\ T_{\text{B}}=\frac{p_{\text{B}}\cdot V_{\text{HT}}}{2\pi }\cdot \sin\frac{\beta }{2}\cdot \sin\left(\delta -\frac{\alpha }{2}-\frac{\beta }{2}\right),\\ T_{\text{T}}=\frac{p_{\text{T}}\cdot V_{\text{HT}}}{2\pi }\cdot \sin\frac{\gamma }{2}\cdot \sin\left(\delta +\frac{\alpha }{2}+\frac{\gamma }{2}\right). \end{array}$$

**Figure 8:**
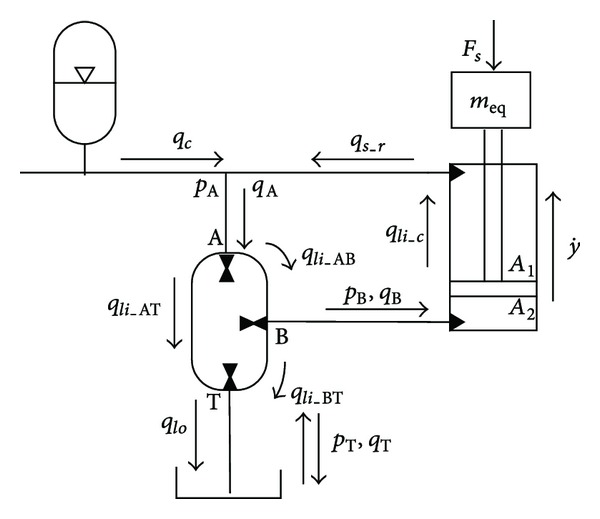
Schematic of SHT control cylinder.

Figure 8 redraws the principle of the SHT control cylinder. Newton's second law is used to obtain the force balance equation. Therefore,

(19) $$\begin{array}{c} p_{\text{B}}\cdot A_{2}-p_{\text{A}}\cdot A_{1}=m_{\text{eq}}\cdot g+F_{f}+F_{L}+m\cdot \ddot{l}+B_{v}\cdot \dot{l}, \end{array}$$

where *A*_1_ and *A*_2_ are the areas of the arm cylinder, *m* is equivalent mass of the arm and load, *l* presents the displacement of arm cylinder, *F*_*f*_ is friction force, and *B*_*v*_ is a viscous damping coefficient, and *F*_*L*_ represents the load which is applied on the cylinder.

For the arm cylinder, the flow rate goes into the bore side of cylinder, and moves out of the rod side of cylinder are calculated by

(20) $$\begin{array}{c} q_{s\_b}=A_{2}\dot{l}+L_{\text{ic}}\left(p_{\text{B}}-p_{\text{A}}\right),\\ q_{s\_r}=A_{1}\dot{l}+L_{\text{ic}}\left(p_{\text{B}}-p_{\text{A}}\right), \end{array}$$

where *q*_*s*_*b*_ is the flow rate which goes into the bore side of the arm cylinder from the SHT, *q*_*s*_*r*_ represents the flow rate out from the rod side of the cylinder to the PHP, and *L*_ic_ is the internal leakage coefficient of the arm cylinder. 

For the flow rate of the SHT, the flow goes into the A port and out from the B port of SHT which follows the equations below:

(21) qA=ωHT·VHT2π·sinα2·sinδ+Lis(pA−pB)  +Lis(pA−pT)+LespAqB=ωHT·VHT2π·sinβ2·sin⁡(δ−α2−β2)  −Lis(pB−pA)−Lis(pB−pT)−LespB,

where *L*_is_ is the internal leakage coefficient of the SHT and *L*_es_ means the external leakage coefficient.

After combining the above equations, we can get the pressure build-up equation for the B port of the SHT:

(22) $$\begin{array}{c} {\dot{P}}_{\text{B}}=\frac{1}{\left(1/\beta_{e}\right)\left(V_{l\_b}+A_{2}\cdot l\right)}\left(q_{\text{B}}-q_{s\_b}\right), \end{array}$$

where *V*_*l*_*b*_ is the hydraulic capacitance including the B port of the SHT, on the bore side of the arm cylinder and the pipe volume between them.

#### 4.2.5. The Subsystem Dynamic Model of Motor/Pump

In the HHEC, the rotation load is driven by the motor/pump, and the dynamic equation is

(23) (pa·Vm_max⁡·β2·π−Ts)·i·ηg  =JM_eq·φ¨+TL+fv·φ˙+fc·sign⁡(φ˙),

where *p*_*a*_ is the pressure of the PHP, and *V*_*m*_max⁡_ is the maximum displacement of the motor/pump; *η*_*g*_ represents the mechanical efficiency of the gear box; *J*_*M*_eq_ is the equivalent moment of inertia of cabin.

#### 4.2.6. The Pressure of PHP

The PHP contains a main pump, hydraulic accumulator, and the actuators. The pressure of the PHP is calculated by the following equation, and it is noticed that again travel motors are omitted in the part:

(24) P˙A=(Qp−∑i=13Qi_HT_A−Qm+∑i=13Qi_r−QL)×((1βe)[∑i=13Vi_a+Vm_a+∑i=13Ai_1·(Hi_sk−li)]  +Caccu)−1,

where *i* represents the index of each actuator, such as bucket, arm, and boom cylinder; ∑_*i*=1_^3^ *V*_*i*_*a*_ is the total capacity which includes each A port of the SHTs, every cylinder volume of the rod side, and the pipe line volume; the initial volume of the motor/pump is represented as *V*_*m*_*a*_; *H*_*i*_sk_ is the stroke of each cylinder and *l*_*i*_ is the displacement of every cylinder; *Q*_*p*_ represents the output flow rate of the main pump; *Q*_*i*_HT_A_ is the A port flow rate of the SHT; *Q*_*m*_ is the flow rate which goes into the motor/pump; *Q*_*i*_*r*_ is the rod side flow rate of each cylinder, and *Q*_*L*_ is the total flow rate of leakage.

Also, in the above equation, *C*_accu_ is defined as the capacity of the accumulator which is the function [32]:

(25) $$\begin{array}{c} C_{\text{accu}}=\frac{V_{a}}{k}{\left(\frac{P_{\text{pre}}}{P_{\text{A}}^{k+1}}\right)}^{1/k}. \end{array}$$

## 5. Control Strategy and Simulation Model

The controller design includes two aspects: the first one is in order to accomplish the speed control by regulating the swash plate angle and the port plate angle of the SHT, and the other is by controlling both engine speed and the displacement of the main pump to reduce the fuel consumption. However, recalling the object of the paper is to study the fuel consumption of the HHEC under the condition which maintained the performance. Hence, to make the comparison fairly, we still adopt the previous constant speed control for the engine which is used universally among construction machinery. It means that the speed governor will regulate the throttle when load changes so as to keep the engine speed constant. Therefore, the controller design for the second part will focus on the displacement control of the main pump.

**Figure 9:**
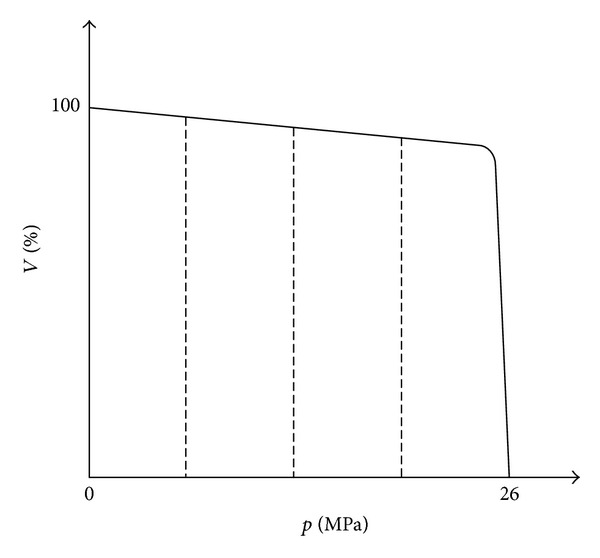
Relationship between pressure and setting displacement of main pump.

*Rule  1.* The highest pressure control is used which limits the maximum pressure at the pump outlet within the pump's control range, which is showed by Figure 9. It means when system pressure exceeds the setting pressure, the displacement of the main pump will reduce so as to avoid the reliving loss and keep system pressure constant. The relationship between pressure and displacement is input into the whole simulation model according to the manufacture document.

**Figure 10:**
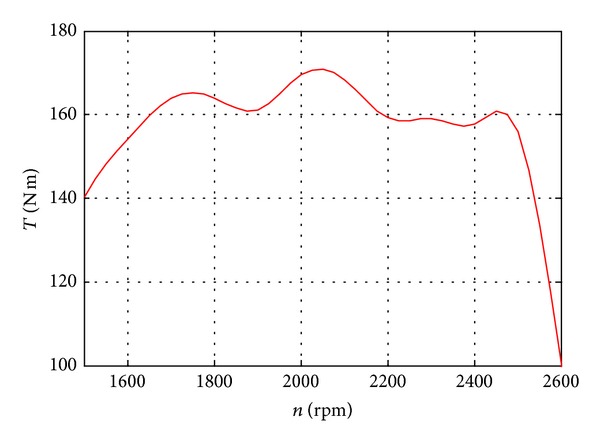
Maximum output torque of engine.

*Rule  2*. To avoid shutting down the engine due to the slow dynamic response, the maximum displacement of the main pump is restricted according to the maximum torque under different engine speed. So the maximum displacement of the main pump *V*_main_max⁡_ is calculated by Figure 10 which shows the maximum torque and the equation below, and the engine data is from [35] as follows:

(26) $$\begin{array}{c} V_{\text{main}\_\max}=\frac{T_{\max\_\text{speed}}-T_{\text{loss}}}{2\pi \cdot p_{\text{A}}}\cdot \eta . \end{array}$$

In the above equation, *T*_max⁡_speed_ is the maximum torque which is obtained by Figure 8, and the engine speed is obtained from the sensor; *η* is the safe coefficient and in this setting the value is 0.9.

**Figure 11:**
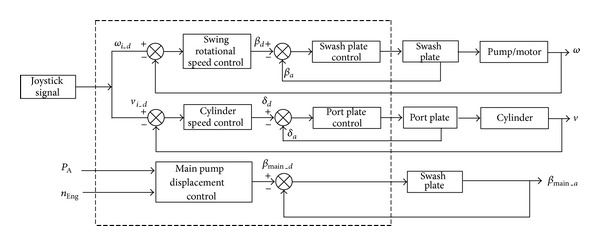
Control principle of HEEC.

The final command value of the displacement of the main pump is to compare two rules and choose the smaller one. The rules are integrated in the module “main pump displacement control”, and the whole control flow chat is plotted in Figure 10.  Figure 11 also shows the speed control principle of the HEEC; the deviation of joystick command speed and the real actuators speed signals are used to control the angle of the swash plate or port plate. Only one control circle for the SHT control cylinder is drawn in Figure 10 for clarity; however, there are three circles which include the boom, arm, and bucket in the real controller. 

## 6. Simulation Results

**Figure 12:**
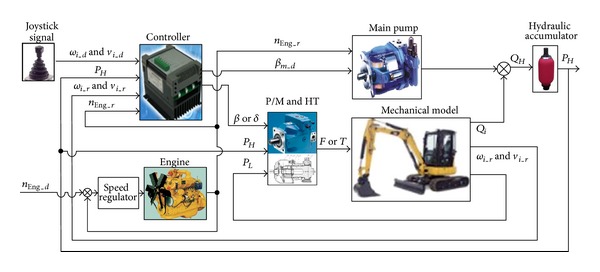
Top module of the whole simulation model.

The whole simulation model is constructed according to the equations above and built by the Simulink software. In addition, the mechanical model which is designed by software simulation X is integrated, and Figure 12 shows the top module lever of the model. A typical excavator digging cycle was selected for simulation which means an excavator digging a bucket of dirt, rotating, and releasing the dirt onto a pile, finally, returning to its initial position. The simulation date is from reference [36]. In order to make a precise simulation, we choose the same procedure with it, and the measurement speed is input into the simulation model as the command speed, and at the same time the load is also input into the mechanical model which is corresponding to the measurement speed.

### 6.1. Speed Response of Each Actuator

**Figure 13:**
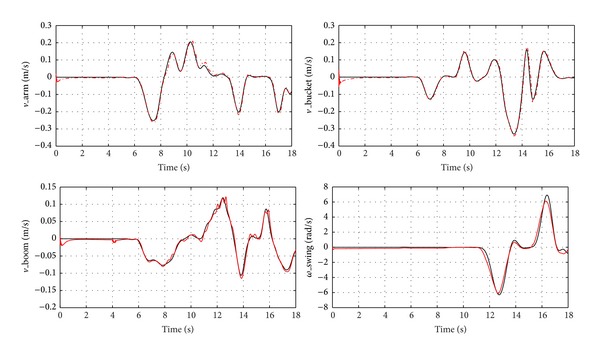
Speed response of each actuator.

**Figure 14:**
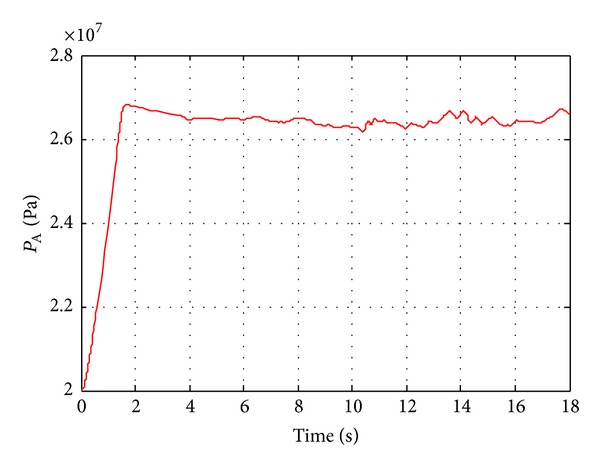
Pressure change of PHP.

The speed response of each actuator is shown in Figure 13, and the red curve is real speed; the other one is command. It can be found that the actuator speed can track the speed command and the performance is acceptable for engineering purposes. However, two problems could be pointed out: one is that the speed oscillation during the initial part. This is because it needs time for pressure build-up of the process. This issue can be addressed by adding a pilot check valve to hold the load during the pressure build-up. Furthermore, speed fluctuates substantially when the load changes violently. The problem is because we only use the simple PI control for the speed control system. The performance can be improved by using an advanced control algorithm. This is also one of the main topics for future work. Moreover, the pressure change of the PHP is plotted in Figure 14.

We can find that the pressure of the PHP ranges from 26 to 27 MPa (10^6^ Pa), and recall that the setting pressure of main pump is 26 MPa. It means that the PHPE is a quasi-constant pressure system which is beneficial to enhance the control performance of the actuators. There are two main reasons for the quasi-constant system pressure. The first one is for the high power of the engine which can keep the displacement of the pump large enough to the supply flow rate. The other reason is because of the accumulator. It can increase the capacity of the PHP so as to balance the flow rate. Actually, the second reason is also influenced by the first since it will also reduce the engine power, but the original engine power seems higher. Of course, this kind of sizing and control strategy is not optimal. So the long-term future objective of this project is to reduce the fuel consumption by reducing the engine power. Then the engine and accumulator can work together; for example, the main pump and accumulator will supply the flow rate together when the system requirement is large; for example otherwise, the redundant flow rate can be stored in the accumulator. For this setting, the smaller engine can work around the minimum fuel consumption area. 

### 6.2. Fuel Consumption of HEEC

**Figure 15:**
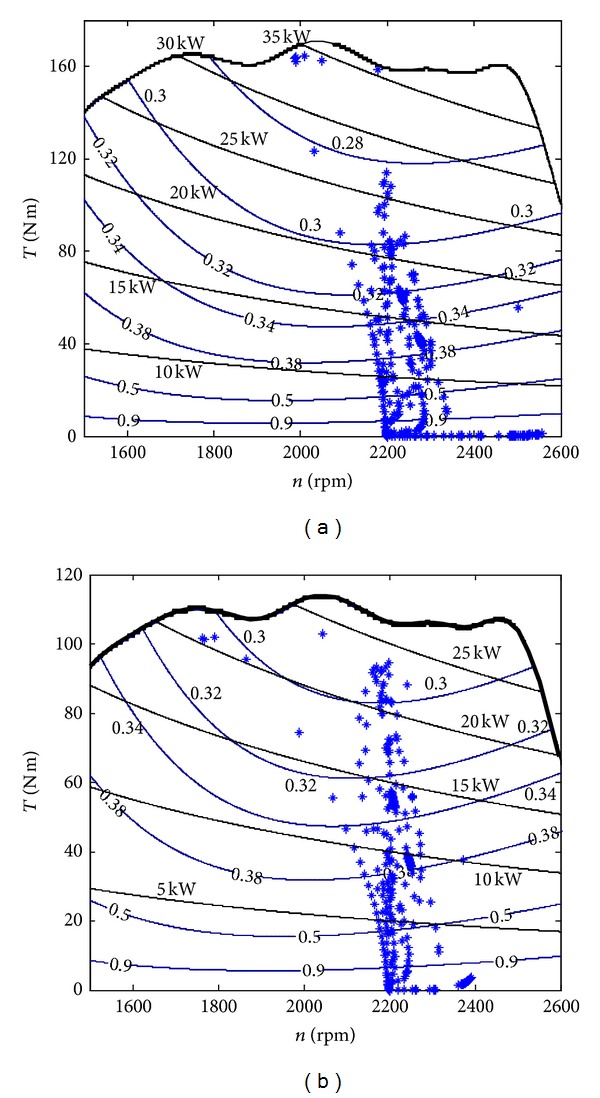
Engine operating point during cycle.

According to Figure 15(a), we can know that the speed of engine can run around 2200 rpm and the torque is less than the maximum torque which can avoid engine shutdown. However, the majority of them are located in the small power area. This means that the HHEC can balance the system power requirement, and the rated engine power can be reduced. Recalling the objective of this paper is to compare the fuel consumption fairly, so the engine power is not chosen and smaller than the original. Then, we choose one smaller engine whose power is 25 kW and the fuel consumption areas are reduced by a scale, too. The simulation is run again and the result is plotted by Figure 15(b). It can be seen that the engine operating points distribute more uniformly. All of the three settings are listed in Table 1. The result shows that the fuel consumption can be reduced 21% compared with the original system and 32% after reducing the rated engine power. 

It needs to be noticed that in the whole cycle, there are no actuator movement commands during the first 6 s, and the main engine power is used to charge the accumulator. So, with the more cycles' work, the average fuel consumption can be reduced further. Hence, it can be expected that the average fuel consumption can be reduced further after running more cycles. Of course, the engine operating points do not lie in the optimal fuel consumption area which should be located along with the line tangent of equal power contour line and equal fuel consumption contour line. So the future objective of this project is to finish the optimal control by controlling engine speed and displacement of the main pump simultaneously. 

## 7. Conclusion

This paper focuses on the fuel consumption of the hydraulic hybrid excavator based on CPR under the precondition of maintaining the control performance. After introducing the principle of CPR and the hydraulic transformer which we have designed, the sizing procedure is finished. Then, a comprehensive excavator model including mechanical, hydraulic, and control systems has been developed by using Simulink. Simulation results show that the new speed control system has a good servo capability. However, it is also necessary to study some advanced control method to enhance the actuator control performance. Moreover, the fuel consumption is reduced 21% compared with the original system, and the result also can be even reduced 32% after adopting one smaller engine. The reasons for improving the fuel consumption are due to the elimination of metering losses and the ability to recover the added power. It is also necessary to notice that the source for reduced fuel consumption is from the operating point of the engine, so the future research will also consider control of the engine's operating point so as to minimize fuel consumption. 

## Figures and Tables

**Table 1:** Fuel consumption values of different system settings.

System type	Engine rated power (kW)	Fuel consumption (g)	Fuel saving ratio (compared with the LS system)
LS system	35	45	—
HEEC	35	35.6	21%
HEEC	25	20.5	32%
